# Age, gender, functional KSS, reason for revision and type of bone defect predict functional outcome 5 years after revision total knee arthroplasty: a multivariable prediction model

**DOI:** 10.1007/s00167-019-05365-x

**Published:** 2019-01-28

**Authors:** Jan F. M. Verbeek, Gerjon Hannink, Koen C. Defoort, Ate B. Wymenga, Petra J. C. Heesterbeek

**Affiliations:** 1 Department of Orthopaedics, Sint Maartenskliniek, P.O. Box 9011, 6500 GM Nijmegen, The Netherlands; 2 Department of Operating Rooms, Radboud University Medical Center, PO Box 9101, 6500HB Nijmegen, The Netherlands; 3 Research Sint Maartenskliniek, P.O. Box 9011, 6500 GM Nijmegen, The Netherlands

**Keywords:** Total knee arthroplasty, Revision, Knee Society Score, Functional outcome, Clinical prediction model

## Abstract

**Purpose:**

The number of revision total knee arthroplasties (rTKA) is increasing. Unfortunately, not all patients benefit from revision surgery. The aim of this study was to develop a clinical prediction model that can be used to predict the functional outcome 5 years after rTKA.

**Methods:**

Data of patients receiving rTKA at Sint Maartenskliniek, Nijmegen, The Netherlands, from 2004 onwards were prospectively collected. Demographic and clinical variables and patient-reported outcome scores were collected and considered as potential predictors. Beneficial outcome was defined as an increase of ≥ 20 points on the functional knee society scores (fKSS) or an absolute fKSS ≥ 80 points 5 years after surgery. The prediction model was developed using backward logistic regression. Regression coefficients were converted into an easy to use prediction rule.

**Results:**

Overall, 295 rTKA patients were included of whom 157 (53%) had beneficial fKSS 5 years later. Age, gender, femoral bone defects, preoperative fKSS, and stiffness as reason for revision were included in the model. Men had a higher chance of beneficial fKSS than women (OR 1.59, 95% CI 0.91–2.78). Patients with major bone defects (OR 0.44, 95% CI 0.22–0.85), higher age (IQR OR 0.39, 95% CI 0.26–0.58), higher preoperative fKSS (IQR OR 0.42, 95% CI 0.30–0.59), and severe stiffness (OR 0.48, 95% CI 0.20–1.15) had a lower chance of successful outcome. The model’s AUC was 0.76, 95% CI 0.70–0.81.

**Conclusion:**

Easily determinable characteristics of patients who need rTKA can be used to predict future functional outcome. Young men with low preoperative fKSS without severe stiffness are more likely to achieve a beneficial outcome.

**Level of evidence:**

IV.

## Introduction

Worldwide, the number of total knee arthroplasties (TKA) is increasing. In the United States, currently more than 700,000 TKA are carried out each year [15]. Likewise, the annual number of TKA revisions has increased from 38,000 in 2005 to 55,000 in 2010, and is expected to grow even further six-fold in the year 2030 [15–18].

The aim of a revision TKA (rTKA) is to provide stable fixation, reduce pain, and improve functional outcome. rTKA is technically a more difficult operation than primary TKA, and is associated with higher risks of complications (28–49%) [2, 30]. Some patients, however, do not benefit from rTKA or even experience a worse outcome, with major negative impact on their quality of life [28, 35]. To overcome these problems, patients should be clearly informed about what they could expect from this operation. Tools have been developed that predict failure of primary TKA [26], but no tool is available to manage patient’s expectations regarding rTKA.

A number of patient-related factors have been identified that play an important role in predicting rTKA outcome, such as age, race, body mass index, and diabetic status [9, 10, 22, 23, 26]. In addition, severe stiffness (formerly referred to as arthrofibrosis) as main reason for revision resulted in a worse outcome compared to other reasons for revision like instability, infection and malposition [33]. Prediction of functional outcome after rTKA might provide insights enabling better management of patient’s expectations. Based on the literature, we hypothesized that predictors for the functional outcome 5 years after rTKA would include age, gender, preoperative patient-reported outcome scores, reason for revision, and type of bone defect. The purpose of this study was to develop a risk prediction model to best predicting functional outcome 5 years after rTKA.

## Materials and methods

Characteristics, surgical details, and outcomes of all rTKA patients treated at the Sint Maartenskliniek, Nijmegen, the Netherlands, were collected in a prospective database from 2004 onwards. We selected patients with a total system revision treated with a condylar stemmed implant (patella could be resurfaced or not, based on surgeon’s decision), and who had at least 5 years of follow-up after rTKA. Excluded were patients with a hinged arthroplasty, revision of unicondylar arthroplasty, patients with relatively small intervention revisions, such as isolated polyethylene exchange and patellar resurfacing, and patients with shorter follow-up than 5 years.

### Surgical technique

All rTKA operations were performed by five orthopaedic surgeons with at least 10 years of experience in the field of revision knee arthroplasty (approximately 25 revisions per year). The surgical procedures used the standard medial parapatellar approach [1]. The cementing or hybrid fixation technique was used for the revision system, and all revision prostheses in this study were posterior stabilized or of the constrained type, including the Genesis II© or Legion© revision system (Smith & Nephew, Inc, Memphis, TN, USA). Routinely, six perioperative microbiological culture samples were taken from the femoral and tibial interface during surgery to diagnose possible septic revisions. Infected TKA were revised in two stages. The first stage involved removal of all components and cement, aggressive surgical debridement, pulsed lavage and ultimately placement of a temporary antibiotics loaded spacer. During the second stage (when infection was treated successfully) the revision components were placed. Tibial wedges and femoral augments were used to restore bone loss. All patients received standard postoperative rehabilitation [33].

### Measurements

#### Outcome measure

The functional knee society score (fKSS) was modeled as outcome measure [12]. The KSS was assessed by the orthopaedic surgeon or resident during regular follow-up visits prior to the revision surgery, at 3 months following surgery, and at 1–2–5 years post-TKA revision. A KSS subscale score of < 60 points was characterized as poor, 60–69 points as fair, 70–79 points as good, and ≥ 80 points as excellent [5]. In standard literature, the minimal clinically important difference (MCID) is defined as the minimal change on a score that is important to the patient. For patients after a primary total or unicompartmental knee arthroplasty, the MCID ranged between 6.1 and 34.5 points [8, 13, 19]. Successful treatment thresholds of fKSS ranged between 72.5 and 85.5 1 year after TKA [13]. As the MCID estimates for the fKSS in rTKA has not yet been identified, we defined a beneficial outcome 5 years after rTKA as an improvement of ≥ 20 points in fKSS, which is similar to a two categories increase (from poor to good/excellent outcome) in the fKSS scale, or an absolute threshold of fKSS ≥ 80 points (excellent outcome).

### Potential predictive factors

Potential predictive factors included age, gender, femoral and tibial bone defects, reason for revision, comorbidity, and preoperative pre-revision scores such as visual analog scale for pain (VAS pain), range of motion, fKSS and cKSS. Comorbidity was assessed using the American Society of Anesthesiologists score (ASA score) [4]. Severe stiffness as a reason for revision was scored positive in patients with limited range of motion (< 70°), with or without pain [33]. Bone defects evaluated during surgery were scored with the Anderson Orthopaedic Research Institute (AORI) classification system, and dichotomized to type I or IIa versus IIb or III [6].

The study protocol was assessed by the regional medical ethical committee [CMO Arnhem–Nijmegen (no. 2003/173)]. Ethical approval was waived by the medical ethical committee on basis of the Dutch medical research involving human subjects act (WMO). The present study has been performed and reported according to the TRIPOD statement for the reporting of multivariable prediction models [3].

### Data analysis

Descriptive statistics were used to summarize the data. Data were missing for four of the seven potential predictive variables and the outcome, ranging between 0.3–10% (Table 1). These missing values were imputed using multiple imputation with the chained equations procedure (predictive mean matching) [32]. Missing data were assumed to be missing at random (MAR), and ten imputed datasets were created.

**Table 1:** Characteristics of patients at baseline for revision TKA and outcome at 5 years postoperative

Characteristic	Total sample (*n* = 295)		Patients with successful functional KSS^b^ (*n* = 157)		Patients with unsuccessful functional KSS (*n* = 119)	
	*n* (%)	Missings *n* (%)	*n* (%)	Missings *n* (%)	*n* (%)	Missings *n* (%)
Gender	295	–	157	–	119	–
Female	198 (67.1)		95 (60.5)		87 (73.1)	
Male	97 (32.9)		62 (39.5)		32 (26.9)	
Age (years)^a^	65 (36–84)	1 (0.3)	63.0 (36–84)	–	67.0 (42–83)	1 (0.8)
KSS subscale^a^						
Function	50 (− 20 to 90)	11 (3.7)	40.0 (− 20 to 90)	3 (1.9)	50.0 (−10 to 90)	–
Clinical	52 (− 14 to 95)	11 (3.7)	52.0 (− 8 to 90)	5 (3.2)	52.0 (− 14 to 95)	2 (1.7)
VAS pain score^a^	64 (0–100)	20 (6.8)	63.5 (0–100)	9 (5.7)	65.5 (0–100)	7 (5.9)
Reason for revision	295	–	157	–	119	–
Aseptic loosening	76 (25.8)		42 (26.8)		30 (25.2)	
Infection	60 (20.3)		35 (22.3)		20 (16.8)	
Instability	52 (17.6)		27 (17.2)		22 (18.5)	
Malposition	77 (26.1)		40 (25.5)		32 (26.9)	
Severe stiffness	30 (10.2)		13 (8.3)		15 (12.6)	
Femoral bone defect	270	25 (8.4)	141	16 (10.2)	111	8 (6.7)
Type I/IIa	211 (78.1)		117 (83.0)		81 (73.0)	
Type IIb/III	59 (21.9)		24 (17.0)		30 (27.0)	
Tibial bone defect, type IIb/III	266	29 (9.8)	137	20 (12.7)	111	8 (6.7)
Type I/IIa	240 (90.2)		124 (90.5)		102 (91.9)	
Type IIb/III	26 (9.8)		13 (9.5)		9 (8.1)	
ASA	275	20 (6.8)	146	11 (7.0)	112	7 (5.9)
ASA I	126 (45.8)		72 (49.3)		46 (41.1)	
ASA II	93 (33.8)		46 (31.5)		41 (36.6)	
≥ ASA III	56 (20.4)		28 (19.2)		25 (22.3)	
*Outcomes 5 years postoperative*						
KSS subscale^a^						
Function	60 (− 20–100)	11 (3.7)	80.0 (10–100)	–	50.0 (− 20–75)	–
Clinical	80.5 (5–100)	29 (9.8)	90.0 (5–100)	13 (8.3)	67.0 (30–100)	8 (6.7)

^a^Data presented as median (range)

^b^Successful functional outcome is defined as a fKSS ≥ 80 or an increase in fKSS ≥ 20 5 years postoperative. There were 19 patients with a missing outcome

Logistic regression was used to evaluate the association between each predictive factor and the outcome. Potential predictors were entered into a logistic regression model taking into account the multiple imputed datasets. Multivariable logistic regression with a backward selection procedure was applied to achieve the most informative and parsimonious combination of predictors. Akaike’s information criterion (*p* < 0.157) was used as selection criterion [36]. The multivariable odds ratios calculated from the logistic regression analysis served evaluating their individual contribution.

The probability (*P*) of having a beneficial functional outcome was constructed with the following formula:

$${P}_{\text{beneficial} \,\text{functional}\, \text{outcome} }=\frac{{e}^{({\beta }_{0}+{\beta }_{1}*{x}_{1}+{\beta }_{2}*{x}_{2}+\dots +{\beta }_{n}*{x}_{n})}}{1+{e}^{({\beta }_{0}+{\beta }_{1}*{x}_{1}+{\beta }_{2}*{x}_{2}+\dots +{\beta }_{n}*{x}_{n})}},$$

where *β*_*0*_ represents the constant, and *β*_*1*_, *β*_*2*_ and *β*_*n*_ the regression coefficients of the predictors *x*_*1*_, *x*_*2*_, and *x*_*n*_, respectively.

Model performance was assessed with calibration plots. The model’s ability to discriminate between patients with successful and unsuccessful outcomes was estimated by the area under the curve (AUC) of the receiver operating characteristics (ROC) curve of the model. Prediction models derived with multivariable regression analyses are known for overfitting, which results in too extreme predictions when applied in new cases. Therefore, the model was validated internally using bootstrapping techniques for which 500 samples were drawn with replacement from the development sample. Bootstrapping techniques provide information on the performance of the model in comparable datasets and generate a shrinkage factor to adjust the regression coefficients and the intercept. After this adjustment, the model performance was reevaluated. No formal sample size calculation was performed. When multivariable prediction models are developed, the sample size is often based on the ratio of the number of individuals with the outcome event to the number of candidate predictors (more precisely, the number of parameters), referred to as the events per variable (EPV). On the basis of some empirical investigations a rule of thumb to have at least 10 EPV was suggested that has been widely adopted [3]. According to this rule of thumb, we could consider a maximum of 11 variables (119 outcome events/10 EPV).

A nomogram was created to easily calculate the probability of having a beneficial functional outcome 5 years after rTKA for a given patient. Statistical analyses were performed using SPSS [version 22 (IBM, New Jersey, US)] and R [version 3.4.3 (R Foundation, Vienna, Austria)] with package ‘rms’.

## Results

The 295 analyzed patients had a median age of 65 years (range 36–84), and were mainly female (198; 67.1%). The median preoperative fKSS score was 50 (range − 20 to 90); the median 5-year postoperative score was 60 (range − 20 to 100). A beneficial fKSS outcome 5 years after rTKA (≥ 20 fKSS improvement or absolute fKSS ≥ 80 values) was observed in 157 patients (53%). The preoperative and postoperative KSS scores, ROM, VAS and the other baseline characteristics are presented in Table 1.

### Predictive ability

After application of the backward selection procedure, the following variables remained in the multivariable prediction model: age at surgery, gender, femoral bone defects, preoperative fKSS, and severe stiffness as reason for revision (Table 2).

**Table 2:** Risk profiles for successful functional KSS 5 years after revision TKA

Predictors	Functional KSS	
	Regression coefficient (β) after internal validation	Odds ratio (95% CI)
Intercept	6.90	–
Age at surgery^a^	− 0.07	0.39 (0.26–0.58)
Gender		
Male	REF	REF
Female	− 0.44	0.63 (0.36–1.10)
Preoperative fKSS^a^	− 0.03	0.42 (0.30–0.59)
Severe stiffness as reason for revision		
No	REF	REF
Yes	− 0.68	0.48 (0.20–1.15)
Femoral bone defect		
Type I/IIa	REF	REF
Type IIb/III	− 0.77	0.44 (0.22–0.85)

^a^Odds ratios (OR) for continuous predictors are presented as inter-quartile range OR

**Fig. 1:**
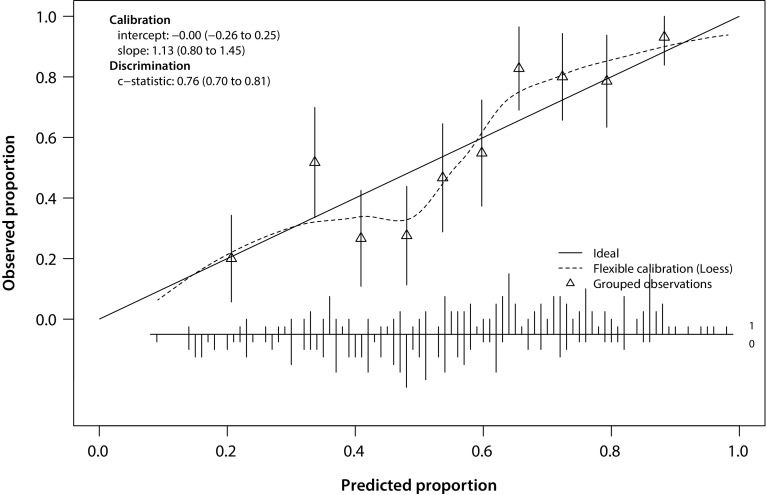
Calibration plot of the prediction model for beneficial fKSS outcomes fitted to the individual data of 295 patients. The triangles represent the observed percentages versus the predicted probabilities of responding. The vertical lines represent the 95% CI of the observed probabilities. The broom plot at the bottom shows the distribution of predicted probabilities separately for patients with and without a beneficial functional outcome

The area under the ROC curve determining how well the model distinguished between patients with beneficial and non-beneficial fKSS 5 years after rTKA was 0.76, 95% CI 0.70–0.81. Through bootstrapping a shrinkage factor of 0.84 was found. After multiplying the regression coefficients with this factor and subsequently adjusting the intercept, the model’s performance was reevaluated. The AUC after internal validation for fKSS was 0.76, 95% CI 0.70–0.81, indicating a good discrimination. Graphical assessment of the model’s calibration after internal validation is shown in Fig. 1.

Male patients had a higher chance of a successful functional outcome 5 years after rTKA surgery than female patients (OR 1.59, 95% CI 0.91–2.78). Patients with bone defects AORI Type IIb/III (OR 0.44, 95%CI 0.22–0.85) patients of higher age (interquartile range OR 0.39, 95% CI 0.26–0.58), patients with higher preoperative fKSS (interquartile range OR 0.42, 95% CI 0.30–0.59), and patients with severe stiffness as reason for revision (OR 0.48, 95% CI 0.20–1.15) had a lower chance of a successful outcome (Table 2).

The probability of having a beneficial functional outcome can be calculated as follows using the regression coefficients presented in Table 2:

$${P}_{\text{beneficial}\, \text{functional} \,\text{outcome} }=\frac{{e}^{\left(lp\right)}}{1+{e}^{\left(lp\right)}}$$

where *lp* = 6.90 + (− 0.07 × age at surgery) + (− 0.44 × female) + (− 0.03 × preoperative fKSS) + (− 0.68 × severe stiffness) + (− 0.77 × Type IIb/III femoral bone defect).

**Fig. 2:**
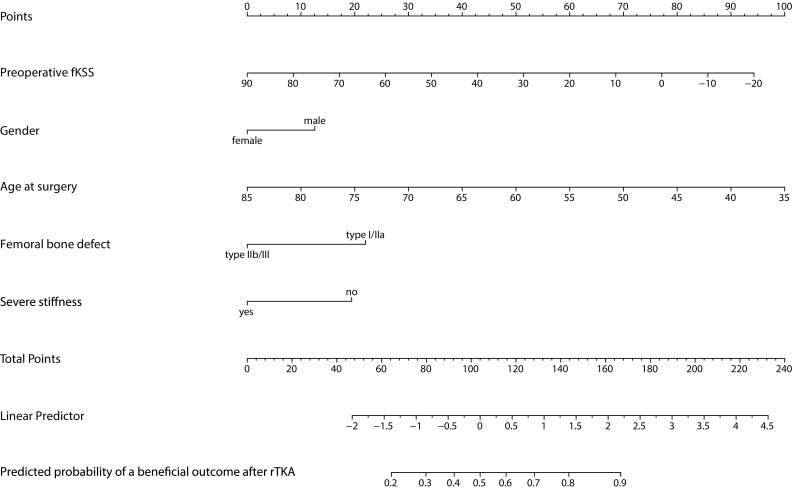
Nomogram for prediction of a beneficial functional outcome 5 years after rTKA in a given patient. To obtain the predicted probability of a beneficial outcome, (1) Locate the patient’s values for “Preoperative fKSS”, “Gender”, “Age at surgery”, “Femoral bone defect”, and “Severe stiffness” on the corresponding axes, (2) Draw a vertical line from the located value on each axis to the “Points”-axis to determine how many points are attributed for each predictor, (3) Sum the points for all variables, (4) Locate the sum on the “Total Points”-axis, and (5) Draw a vertical line towards the “Predicted probability of a beneficial outcome after rTKA”-axis to determine the probability of a beneficial functional outcome 5 years after rTKA. To illustrate the use of the nomogram, the outcome for a 65-year-old female patient with a preoperative fKSS of 50 who has a type IIb AORI femur bone defect, and severe stiffness as main reason for revision is predicted. After locating the patient’s values for “Preoperative fKSS” (50), “Gender” (female), “Age at surgery” (65), “Femoral bone defect” (type IIb), and “Severe stiffness” (yes) on the corresponding axes, draw a vertical line up from each of these values to the “Points”-axis to determine how many points are attributed for each predictor. “Gender” (female), “Femoral bone defect” (type IIb), and “Severe stiffness” (yes) are attributed 0 points each. “Preoperative fKSS” (50) and “Age at surgery” (65) are attributed 34 and 39 points, respectively. The sum of the attributed points is 73. After locating 73 on the “Total Points”-axis, a vertical line is drawn downwards to the “Predicted probability of a beneficial outcome after rTKA”-axis to determine the probability of a beneficial functional outcome 5 years after rTKA, which is 26% for this particular patient

The nomogram created as a tool to easily calculate the probability of having a beneficial functional outcome 5 years after rTKA for a given patient is shown in Fig. 2.

### Clinical example

To illustrate the prediction rule we will predict the outcome for a 65-year-old female patient with a preoperative fKSS of 50 who has a type IIb AORI femur bone defect, and severe stiffness as the main reason for revision. We can calculate the predicted probability of a beneficial fKSS after 5 years after rTKA calculated with the formula presented above or using the nomogram (Fig. 2). The predicted probability for this patient is 26% (95% CI 17–35%), i.e., a small chance of a successful fKSS outcome after 5 years. With this information, the patient is provided with more realistic expectations regarding the revision surgery and its late-anticipated poor results. An otherwise good candidate would be a healthy 55-year-old man with preoperative fKSS of 50 points, type IIa AORI femur bone defect, and a different reason than severe stiffness (e.g. infection) for his revision surgery. His probability of a successful fKSS 5 years after TKA revision is 82% (95%CI 75–90%).

## Discussion

The most important finding of this study was that the functional outcome 5 years after rTKA could be adequately predicted using preoperative fKSS, age, gender, stiffness as a reason for revision and the type of femoral bone defect. Combining these factors into a relevant prognostic profile will enable physicians to help manage patients’ expectations about revision surgery.

Preoperative fKSS was inversely related to successful 5-year postoperative fKSS outcome, which was earlier described for a 2-year follow-up period [33], and women had a worse outcome than men after a rTKA. This might be because women have higher rates of obesity even morbid obesity, postoperative transfusion, and extended length of stay, all of which are correlated with poor functional and clinical outcomes [34]. However, in a small retrospective study, no differences in gender were found [27]. The coexistence of major femoral bone effects was associated with worse fKSS outcome. This is in line with a study that used bone allograft to fill the bone defects, whereby 10% of all revision required a re-revision [7]. Unfortunately, bone defects can only be adequately scored perioperatively. Nevertheless, patients’ expectations could adequately be managed preoperatively by providing fKSS estimations with or without major femoral bone defects. In our study, the majority of patients had minor femoral bone defects.

While the minimum clinically important difference for revision surgery is unknown, a definition of beneficial outcome of ≥ 20 for fKSS was used. This definition is in the range of MCIDs reported for primary TKA [8, 13, 19]. In addition, ≥ 80 fKSS was set as a clear threshold to ensure patients who already have a high preoperative KSS score to be scored as a successful treatment [13]. Despite this threshold, most benefits was achieved in patients with poor preoperative fKSS. This is in contrast to a previous smaller study, which did not include a proper MCID and did not adjust for severe stiffness as a reason for revision [14]. Patients with severe stiffness had a poor fKSS outcome in our cohort. Infection was not found to be a predictor for inferior outcomes, which might be surprising. It has been shown that infection as the reason for revision was associated with an inferior functional outcome 2 years after surgery [33]. However, Meek et al. [20] reported that revision operation for infection was associated with reasonable function and satisfaction scores at a mean follow-up of 41 months (minimal follow-up of 2 years). In addition, Patil et al. [25] compared the clinical outcomes and patient satisfaction rates of aseptic versus septic revision TKA at a mean follow-up of 40 months (range 24–80 months). They reported that patients undergoing revision for an infected TKA had better functional outcomes compared to those with revision for aseptic reasons. Revision for severe stiffness was associated with the poorest outcomes.

The risk calculation based on the prediction model can aid the shared decision making when considering a revision TKA. Functional and clinical outcomes from the KSS system are both of interest to orthopedic surgeons, although clinical KSS can be difficult to interpret for patients. The fKSS can be understood as an improvement or decline of their walking ability, including climbing stairs and the use of walking aids. The prediction rule provides a probability between 0% and 100% for beneficial KSS outcome 5 years after revision total knee arthroplasty. However, a 50% probability of beneficial outcome might still leave the patient in doubt as to what to expect. It is important to correctly set the indication for revision. For example, if a TKA is infected, this alone would justify a revision, even if the outcome prediction is poor. However, the model can still be used to guide patients’ expectations in these situations.

This study has some potential limitations that need to be discussed. The study was conducted in a relatively young population, with few comorbidities. Differences in case-mix, for instance, may result in introducing new predictors that in turn could further improve the performance of the model and resulting in a better application in daily practice and for general use. To make the model easier to use in daily practice, only easily accessible predictors were included. Furthermore, it would have been preferable to consider other potential predictors such as body mass index, diabetic status, smoking, mental status, and time between primary and revision TKA to be included in the model [21, 24, 29]. However, these variables were not recorded. Evidently, the outcome can also be affected by factors as perioperative findings or complications during surgery, or injuries after surgery. These unknown factors were also not included in our model.

Missing values were imputed using multiple imputation techniques, which further improved the predictive ability of the prognostic model [11, 31]. Sample size was limited, which introduced overfitting. Internal validation with bootstrapping techniques were used to adjust for this overfitting [11, 31].

Patients were evaluated 5 years after surgery. Truncating the follow-up data at 2 years would have increased the number of patients, but at the cost of missing relevant changes in KSS. In addition, a longer follow-up gives patients more relevant expectations regarding the clinical process [23]. Before integrating this prediction rule in daily practice, the prediction rule needs to be externally validated, and important determinants such as BMI, smoking and diabetes mellitus should be taken into consideration as well. In daily clinical practice, prediction of functional outcome after rTKA based on the presented prediction model may help clinicians to enable better management of patient expectations and aid shared decision making when considering a rTKA.

## Conclusion

Future functional outcome among patients who need a revision of their total knee arthroplasty can be predicted using easily accessible patient characteristics. Patients are more likely to achieve a beneficial outcome if they are male, younger, have lower preoperative KSS, and reasons for revision other than severe stiffness. Combining these factors into a relevant prognostic profile will enable physicians to help manage patients’ expectations about revision surgery.

## Data Availability

The datasets generated and/or analysed during the current study are not publicly available but are available from the corresponding author (GH) on reasonable request.
